# Estimating cost savings from regionalizing cardiac procedures using hospital discharge data

**DOI:** 10.1186/1478-7547-5-7

**Published:** 2007-06-29

**Authors:** Vivian Ho, Laura A Petersen

**Affiliations:** 1 Baker Institute, Rice University, 6100 Main Street, Houston, TX, 77005, USA; 2 Department of Medicine, Baylor College of Medicine, Houston, TX, USA; 3 Division of Health Policy and Quality, Houston Center for Quality of Care and Utilization Studies, Veteran Affairs Medical Center, 2002 Holcombe Boulevard, Houston, TX, 77030, USA; 4 Section for Health Services Research Department of Medicine, Baylor College of Medicine, Houston, TX, USA

## Abstract

**Background:**

We examined whether higher procedure volumes for coronary artery bypass graft (CABG) surgery or percutaneous coronary interventions (PCI) were associated with lower costs per patient, and if so, estimated the financial savings from regionalizing cardiac procedures.

**Methods:**

Cost regressions with hospital-specific dummy variables measured within-hospital cost reductions associated with increasing hospital volume. We used the regression estimates to predict the change in total costs that would result from moving patients in low-volume hospitals to higher volume facilities.

**Results:**

A 10% increase in PCI procedure volume lowered costs per patient by 0.7%. For the average hospital performing CABG in 2000, a 10% increase in volume was associated with a 2.8% reduction in average costs. Despite these lower costs, the predicted savings from regionalizing all PCI procedures in the sample from lower to high-volume hospitals amounted to only 1.1% of the entire costs of performing PCI procedures for the sample in 2000. Similarly, the cost savings for CABG were estimated to be only 3.5%.

**Conclusion:**

Higher volumes were associated with lower costs per procedure. However, the total potential savings from regionalizing cardiac procedures is relatively minor, and may not justify the risks of reducing access to needed services.

## Background

The number of patients who received percutaneous coronary interventions (PCI) or coronary artery bypass graft surgery (CABG) in the US has changed dramatically over time. For example, the number of PCI procedures rose from 377,611 to 692,621 between 1993 and 2002, an 83% increase. The number of CABG procedures rose from 312,109 in 1993 to a peak of 383,788 in 1997, before falling to 316,471 in 2002 [1].

Although the overall volume of cardiac procedures has increased, many individual hospitals do very few of these procedures. Several studies have found that low hospital procedure volume is associated with higher mortality, length of stay, and complication rates [2-7]. These results have led to recommendations to regionalize certain procedures [8,9]. Regionalization policies that concentrate care at a smaller number of providers should lead to higher volumes and therefore improved patient outcomes, with little or no consequences for patient travel [10].

However, little is known about the cost implications of regionalizing care. Past studies have yielded contradictory evidence on whether high-volume facilities perform PCI and CABG at lower costs per patient. Some studies find substantial cost savings associated with high procedure volume [11,12]; while other research finds no evidence of a volume-cost relationship [13]. This disagreement may be due to the relatively small sample sizes and short time periods which were used to analyze the association between provider volume and costs. Although recommendations to regionalize care are based primarily on improvement in patient outcomes, the cost implications are significant. If high-volume hospitals operate at lower costs, then this policy would be one of very few interventions that is not just cost-effective, but cost saving.

Our goal was to use nationally representative data to determine whether higher cardiac procedure volumes were associated with lower average costs per procedure and to estimate potential cost savings from regionalizing procedures. We studied all admissions for PCI and CABG from a nationally representative sample of hospitals between 1988 and 2000 to test for a volume-cost relationship.

## Methods

We used data from the Agency for Healthcare Research and Quality (AHRQ) Healthcare Cost and Utilization Project (HCUP) Nationwide Inpatient Sample (NIS) database. The HCUP NIS data is a stratified random sample of community hospitals in the United States. The dataset annually samples 20 percent of acute care hospitals in each of five strata (geographic region, ownership, location, teaching status, and bedsize). The first release of the NIS for the years 1988 to 1992 contained information from 11 US states, and the 2000 release contains information on over 1,000 hospitals in 28 states. On average there are four years of data for each hospital in this study. All inpatients with a procedure code for PCI (including stents) (ICD-9-CM 36.0, 36.00, 36.01, 36.02, or 36.05) or CABG (ICD-9-CM 36.1 x) in any field of the discharge abstract were selected. The sample contained data on 439 different hospitals that performed CABG and 491 hospitals that performed PCI between 1988 and 2000. Sample weights provided in the NIS were applied during the analysis, so that the results are representative of the entire US population who received PCI and CABG.

### Hospital Volume and Costs

The NIS contains all discharge abstracts from each hospital it samples, permitting accurate counts of PCI and CABG volume for each facility. The annual procedure volume for each hospital and year was calculated as the number of discharges with the respective procedure code during the calendar year of admission. Any records for which this process revealed that the hospital performed five or fewer procedures during the year were excluded from the analysis.

The NIS provides total charges for each patient stay in the hospital, which have been edited for excessively low and high charges [14]. These charges were deflated by the All-Urban Consumer Price Index to reflect year 2000 dollars. The charges were then multiplied by a hospital- and year-specific cost-to-charge ratio to reflect the costs of each admission. A cost-to-charge ratio for each hospital and year were derived from annual Medicare cost reports.

The Centers for Medicare and Medicaid Services (CMS) has structured its regulations in an effort to obtain cost and charge data in hospital cost reports that most accurately reflects resource utilization. Hospital cost report data is used to appropriately adjust prospective payment rates for inpatient care to reflect resource utilization. As stated in the Code of Federal Regulations: "Adequate cost information must be obtained from the provider's records to support payments made for services furnished to beneficiaries. The requirement of adequacy of data implies that the data be accurate and in sufficient detail to accomplish the purposes for which it is intended [15]." In particular CMS relies on the cost reports to determine the resource utilization of Medicare beneficiaries versus other hospital patients: "Total allowable costs of a provider will be apportioned between program beneficiaries and other patients so that the share borne by the program is based upon actual services received by program beneficiaries."

CMS relies in part on the charges reported in hospital cost reports to determine Medicare patients' costs relative to all patients. For example, the ratio of Medicare patients' charges to all patients' charges is used to apportion ancillary department costs to revenue-generating departments. CMS has adopted this approach, because "An increasing number of third-party purchasers who pay for services on the basis of cost are developing methods that utilize charges to measure the amount of services for which they have responsibility for payment."

Patients with costs below $1,000, which were likely to reflect coding error, were removed from the sample. Six states in the NIS did not provide hospital identification codes which are needed to merge the NIS with Medicare data, and therefore hospitals in these states were excluded from the analysis.

### Other Study Variables

Age, sex, race, AMI, urgent or emergency admission, and transfer from another hospital were included as explanatory variables in cost regressions. Indicator variables are also included for the individual comorbidities that comprise the Charlson comorbidity index which are: prior AMI, peripheral vascular disease, dementia, chronic obstructive pulmonary disease, rheumatologic disease, liver disease (mild), liver disease (moderate/severe), diabetes (mild/moderate), diabetes with complications, kidney disease, cancer, and metastatic solid tumor. These conditions were coded based on the Dartmouth-Manitoba mapping of the Charlson comorbidity index to ICD-9-CM codes [16]. This mapping recognizes that certain diagnostic and procedure codes may represent complications when they appear on the index discharge abstract (as opposed to abstracts from previous admissions). Therefore, the mapping specifies a subset of ICD-9-CM codes for each condition that excludes potential complications if one only has access to discharge records from the current admission, as we do in this study.

Indicator variables for patients who received multivessel PCI or a stent were included in the cost regressions for PCI patients. We did not include indicator variables for other procedures, such as CABG for patients undergoing PCI. Higher incidence of emergency bypass surgery after PCI has been found in low-volume hospitals [17,18]. Therefore, if patients in low-volume hospitals require more procedures as a result of complications, the regressions attribute the resulting costs to volume.

### Statistical Analysis

We defined low volume PCI as fewer than 200 procedures, medium volume as 200 to 399 procedures, and high volume as 400+ procedures per year. We defined low volume CABG as fewer than 200 surgeries, medium volume as 200 to 449 surgeries, and high volume as 450+ surgeries per year. The cutoff point for high volume hospitals was based on hospital referral guidelines published by the Leapfrog group. Medium volume hospitals were determined by consulting minimum institutional volume recommendations published in the ACC/AHA guidelines as well as past studies [4,8,19-22].

Mean costs are reported for three time periods: 1988–1991, 1992–1996, and 1997–2000. We also report the percentage of patients who are 85 years and over, and the proportion of patients who underwent stent insertion by hospital volume and time period. We hypothesized that both older age and stent insertion contribute to higher costs.

The unit of observation in the regressions is the patient. We estimate separate cost regressions for PCI and CABG. The distribution of patient costs for both CABG and PCI is skewed due to extremely high costs for a small number of outliers. Therefore, the natural logarithm of patient cost was used as the dependent variable in each case in order to reduce the sensitivity of the estimates to extreme observations. In addition to making the estimates more consistent with classical linear model assumptions, the log model allows one to interpret the coefficient estimates as percentage changes in costs as a function of unit increases in the explanatory variables [23].

For both PCI and CABG, there was only one regression for which the association between costs and volume was precisely estimated, and for which alternative specifications (such as adding the square and the cube of hospital volume) yielded no additional explanatory power [24]. The natural log of hospital volume provided the best fit for modeling PCI costs. In contrast, a cubic polynomial of hospital volume in levels provided the best fit for the relation between hospital volume and costs for CABG procedures.

The cost regressions are estimated including dummy variables for each hospital in the sample. This "fixed effects" approach controls for unobservable hospital characteristics that may confound the relationship between volume and costs [25]. For example, one may be concerned that some hospitals may have higher quality facilities that attract more patients, but are also more costly. By including a dummy variable for each hospital, one controls for these systematic differences across hospitals. The regressions therefore provide an estimate of the expected change in costs resulting from a within-hospital increase in procedure volume. The hospital-specific dummy variables will also capture the effects of any observed hospital characteristics which are constant throughout the sample period, such as teaching status. Therefore, the regression coefficients will measure the average size of the relationship between volume and costs over all teaching and non-teaching hospitals in the US This approach is the most reasonable one for a nationwide policy simulation. All regressions were estimated in Stata 8.0, and standard errors were adjusted for the clustering of patients at the hospital level.

Estimates based on within-hospital changes in procedure volume can be used to predict the effects of alternative regionalization policies. For a log (cost) regression where hospital volume is expressed in natural logs, the coefficient on volume provides the percentage increase in costs resulting from a one percentage point increase in hospital volume. To compute a consistent estimate of the percent change in costs for a log(cost) regression where hospital volume is specified in levels and polynomials of volume, we first multiplied each polynomial in volume in the regression by its respective coefficient. We then took the exponential of the sum of these products, subtracted 1, and multiplied the resulting number by 100 [23].

Because the NIS sampled only 20 percent of hospitals in the participating states, one cannot predict the results of closing low-volume hospitals and referring the patients in these hospitals to the geographically closest high-volume hospital. Instead, we used the regression estimates to predict the change in total costs that would result from moving patients in low-volume hospitals to representative higher volume hospitals. For both PCI and CABG, we identified all patients treated in low-volume hospitals in the last year of the sample, 2000. We then predicted the change in costs that would result if they had instead been treated in a "typical" higher volume hospital, defined as a facility with the median procedure volume of all medium and high volume hospitals in the sample in 2000. We also predicted the change in costs that would result if all patients treated in low and medium volume hospitals were instead treated in a hospital with the median size of all high-volume hospitals in 2000. Simulations of the change in costs resulting from closing low-volume hospitals required converting regression estimates so that they reflected an unbiased estimate of costs rather than log (costs) [23].

## Results

The NIS contained data on 1,046,630 PCI admissions and 828,148 admissions for CABG. Six states in the NIS did not provide hospital identification codes which are needed to merge the NIS with Medicare data. For this reason, we excluded 96,791 PCI admissions and 68,254 CABG admissions from the analysis. In addition, 19,503 PCI records and 15,726 CABG records were missing information on charges. There were 9 PCI admissions and 12 CABG admissions that were missing information on patient gender. Our regressions were based on the remaining 930,327 PCI patients and 744,156 CABG patients. These data represent 98 percent of the patients in the states which reported hospital identifiers.

Table 1 illustrates the distribution of both hospitals and patients by hospital procedure volume. The data reveal a gradual increase in high-volume hospitals and a greater propensity for patients to be treated in high-volume hospitals over time. However, by 1997–2000 approximately one quarter of hospitals performing PCI and/or CABG in the sample were still considered to be low-volume facilities. Because by definition these facilities treated fewer patients, less than 10 percent of patients were treated in low-volume facilities for most of the sample period. Yet by the last time period, 1997–2000, 16.1 percent of PCI patients and 37.4 percent of CABG patients were still being treated in hospitals which did not meet the Leapfrog Group's volume-based standard of care.

**Table 1:** Distribution and total number of hospitals and patients* by hospital volume and year

	**PCI**				**CABG**			
	**Hospital Volume**				**Hospital Volume**			

	**< 200**	**200–399**	**400+**	**Total #**	**< 200**	**200–449**	**450+**	**Total #**

**Hospitals**								
1988–1991	41.0%	32.7%	26.3%	806	36.0%	42.1%	21.9%	773
1992–1996^†^	25.5%	28.0%	46.5%	874^†^	28.4%	42.5%	29.1%	829^†^
1997–2000	24.8%	22.4%	52.8%	841	27.6%	39.8%	32.6%	753
**Patients**								
1998–1991	12.9%	29.7%	57.4%	1.03 million	11.3%	39.2%	49.4%	1.06 million
1992–1996^†^	5.4%	17.5%	77.2%	2.03 million^†^	7.9%	33.8%	58.2%	1.67 million^†^
1997–2000	4.2%	11.9%	83.9%	1.90 million	7.9%	29.5%	62.6%	1.27 million

*The data are weighted so that they are representative of the US population.

^†^The middle time period is based on five years of data; other time periods contain data from four years.

### Average Costs per Procedure

Table 2 provides information on the average costs of care for patients who received either PCI or CABG by hospital volume and time period. The average cost in 2000 dollars of performing PCI declined from $12,451 in 1988–1991 to $11,363 in 1997–2000. The average cost of performing CABG declined even further over the sample period, from $30,733 in 1988–1991 to $24,001 in 1997–2000. This pattern is consistent with previous studies reporting declining real costs of performing PCI and CABG between 1984 and 1994 [26]. Within each time period there is also an association between higher hospital procedure volume and lower costs per patient. Table 2 indicates that the percentage of patients age 85 years or older rose throughout the sample for both procedures. The table also reveals the dramatic rise in stent use over the sample period, as well as a positive correlation between hospital volume and stent use.

**Table 2:** Trends in average costs and patient characteristics by hospital procedure and volume 1998–2000

	**PCI**				**CABG**			
	**Hospital Volume**				**Hospital Volume**			

	**< 200**	**200–399**	**400+**	**Total #**	**< 200**	**200–449**	**450+**	**Total #**

Cost*								
1988–1991	$13,599	$12,787	$12,019	$12,451	$37,219	$30,704	$29,271	$30,733
1992–1996	$13,592	$13,043	$11,617	$11,972	$33,241	$28,035	$26,252	$27,409
1997–2000	$12,606	$12,563	$11,130	$11,363	$26,862	$24,293	$23,500	$24,001
**Age 85+, %**								
1988–1991	0.9	1.0	0.9	0.9	0.7	0.8	0.7	0.7
1992–1996	1.6	1.6	1.6	1.6	1.1	1.2	1.2	1.2
1997–2000	3.0	2.9	2.8	2.8	1.8	1.8	2.0	1.9
**Stent, %**								
1988–1991	0	0	0	0				
1992–1996	6.4	7.9	11.4	10.5				
1997–2000	67.4	73.3	76.5	75.8				

*Average cost per procedure in year 2000 dollars.

Each 10 percent increase in a hospital's PCI procedure volume reduces average costs per patient by 0.7 percentage points (p = 0.012), controlling for year, patient characteristics, and hospital fixed effects (Table 3). Mean PCI procedure volume over the sample period rose by 139 percent (from 255 to 610) between 1988 and 2000. This magnitude of increase in procedure volume is predicted to lower average costs per patient by 9.7 percent.

**Table 3:** Regression estimates of determinants of log(average costs per procedure)

	**PCI**		**CABG**	
	**Coefficient**	**CI**	**Coefficient**	**CI**

**Log(volume)**	-0.07	(-0.124– -0.016)		
**Volume**			-0.0007	(-0.0012– -0.0002)
**Volume**^2^			6.62E-07	(1.26E-07– 1.20E-06)
**Volume**^3^			-1.79E-10	(-3.37E-10– -2.16E-11)
**Sample Size**	930,327		744,156	

Regressions also include year dummy variables (1989 to 2000), indicators for age (65–69, 70–74, 80–84, 85 years), female, black race, AMI, urgent or emergency admission, transfer from another hospital, the individual components of the Charlson comorbidity index, multivessel PCI or stent use for PCI patients, and a dummy variable for each hospital in the sample.

The mean CABG hospital procedure volume in the year 2000 in our sample is 405 procedures. A 10 percent increase in CABG volume (41 procedures) is predicted to reduce costs per patient by 2.8 percent. Mean CABG procedure volume over the sample period rose by 82 procedures (from 323 to 405) between 1988 and 2000. This magnitude of increase is predicted to lower average costs per inpatient stay by 5.2 percent. From a baseline cost of $23,910 (the average cost per CABG patient in 2000), a 5.2 percent reduction in costs would equal $1,243.

To determine whether the negative association between volume and costs was consistent for the thirteen years in the study, we re-estimated the regressions in Table 3 including interactions between procedure volume and the two later time periods (1992 to 1996 and 1997 to 2000). For both PCI and CABG, the interaction terms were insignificant, with the lowest p-value being equal to 0.221.

### Estimated Cost Savings of Regionalization Policies

**Figure 1:**
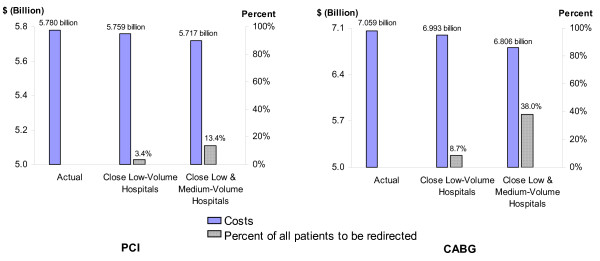
**Actual costs and predicted results of closing lower volume hospitals and redirecting patients to higher volume facilities in 2000**. ^a ^Predictions of total costs and patients affected if patients in low-volume hospitals were instead treated in a hospital with the median procedure volume of medium and high-volume hospitals in year 2000. ^b ^Predictions of total costs and patients affected if patients in low and medium-volume hospitals were instead treated in a hospital with the median procedure volume of high-volume hospitals in year 2000.

We used the regression estimates to predict the change in costs that would result from eliminating PCI and CABG in low-volume hospitals and moving these patients instead to higher volume hospitals (Figure 1). A total of 17,236 PCI patients out of 509,491 (3.4%) were treated in low-volume hospitals in the year 2000. Among medium and high-volume hospitals in the sample in 2000, the median PCI procedure volume was 539. If these 17,236 patients had instead been treated in a hospital which performed 539 PCI procedures per year, the regression estimates indicate that the total costs of caring for these patients would be lowered by $22.1 million.

Altogether 68,020 PCI patients (13.4%) in the year 2000 were treated in low or medium volume hospitals. If these patients had instead been treated in the median high-volume hospital for 2000, which performed 708 procedures, total costs were estimated to be lowered by $63.1 million. The total cost of performing all PCI procedures in the sample for the year 2000 equaled $5.8 billion. Therefore, the predicted savings from regionalizing all PCI procedures to high-volume hospitals amounted to 1.1% of the entire costs of performing the procedure in 2000.

Similarly, if the 25,646 patients (8.7%) in low-volume hospitals had instead been treated in a hospital which performed 400 CABG procedures per year, the regression estimates suggest that total costs would be lowered by $66.4 million; and if patients treated at both low- and medium-volume sites (38.0%) had instead been treated in a hospital with the median high-volume procedure load of 710 procedures in 2000, the total costs of caring for these patients were estimated to be lowered by $252.8 million. The total cost of performing all CABG surgeries for the year 2000 equaled $7.1 billion. Therefore, the predicted savings from regionalizing all CABG procedures to high-volume hospitals amounted to only 3.5% of the entire costs of performing the procedure in 2000.

The number of states in the HCUP increased from 11 to 28 over the sample period [27], so that our estimates may have been affected by sample composition. We re-estimated the cost regressions restricting the sample to hospitals in the 11 states which were represented in all 13 years of the sample. The magnitude of the coefficient estimates remained virtually the same, although the precision of the estimates decreased slightly. We also repeated the regression analysis without the NIS sample weights, which were constructed by the AHRQ to yield results which are representative of the entire US population. The differences in results, if at all, were in the third decimal place of the coefficients reported in Table 3. The minimal differences suggest that the sample is indeed representative of the relationship between volume and costs in the nation as a whole.

## Discussion

Using nationally representative data from the years 1988–2000, we determined that higher cardiac procedure volumes were associated with lower average costs per procedure. This finding was robust and consistent over the thirteen years of our study. Surprisingly, despite these lower average costs, the absolute magnitude of cost savings that could be achieved was relatively small. For example, we found that the predicted savings from regionalizing all PCI procedures in the sample to high-volume hospitals amounted to only 1.1% of the entire costs of performing PCI procedures for the sample in that year. Similarly, the cost savings for CABG were estimated to be only 3.5%.

A handful of other studies have assessed the relationship between volume and costs for CABG and PCI. Although two studies found a negative association between volume and costs for PCI [11,12], one study of CABG found no association between volume and costs [13]. However, the latter study was based on only twelve hospitals. In addition, past studies of the volume-cost relationship analyzed data for 2 years at most, making it difficult to determine if the findings were robust and consistent across years. One study used Medicare data [11], making the generalization of findings to the non-Medicare population of potential concern. Past studies of PCI or CABG volume and costs were cross-sectional in nature. These studies measured the relationship between volume and costs by comparing costs across hospitals that differed in size over a one to two year time period. Thus, the association between volume and costs could be confounded by other attributes that differ systematically across hospitals, such as the specific technologies used to perform PCI and CABG, the costs of health care workers in the local labor market, or differences in patient casemix that generally are not reported in administrative databases.

We overcame these methodological problems in several ways. First, we used a nationally representative sample of hospitals, and we included all procedures performed at sampled hospitals. We studied costs over 13 years to ensure that our findings were consistent and robust. In order to examine how costs change within a given hospital as its volume increased over time, we used a fixed effects model. Inclusion of a dummy variable for each hospital controls for systematic, unobserved differences across hospitals, such as differences in technology, labor costs, patient population, or the manner in which financial information in cost-to-charge ratios is reported. The regression estimates yield the within-hospital change in costs associated with within-hospital increases in volume over time. We believe that these methods overcome many of the problems with prior studies and yield appropriate measures to predict the potential cost impact of regionalization.

Why did we find only limited potential savings, despite lower average costs per procedure with higher volume? The limited savings are because the number of patients being treated in low-volume hospitals has fallen over time. For example, only 17,236 PCI patients out of 509,491 were treated in low-volume hospitals in the year 2000. Our findings confirm speculation that volume-based referral strategies would not greatly reduce direct health care costs [28]. Adding capacity (i.e. operating rooms and beds) at high-volume referral hospitals could lead to higher costs in the short term. Also, administrative costs associated with transferring patients and their records to high-volume hospitals would be created. Lastly, high-volume hospitals may have more market power, perhaps leading them to raise their charges.

Of course, the primary motivation for regionalization policies has not been cost savings, but improving patient outcomes. Several studies have found that low hospital procedure volume is associated with higher mortality, readmission, and complication rates [8,20,29]. These results have led to recommendations that certain procedures should be regionalized [8]. Concentrating care through regionalization policies at a smaller number of providers should lead to higher volumes and therefore improved patient outcomes.

One limitation of our study is that we were not able to estimate the potential years of life gained in the sample by regionalization. We also may not have ascertained all PCI procedures in the later years of the study, as more PCI procedures were performed in the outpatient setting. However, we sought to study the effect of hospital volume on inpatient costs. It is possible that expertise gained from performing additional outpatient procedures has a "spillover" effect, leading to reduced inpatient PCI costs as well. In this case, inpatient PCI volume yields an underestimate of total procedure volume, and our regressions overestimate the association between increased procedure volume and lower costs. If so, the estimated cost savings from regionalization may be even smaller.

Despite the efforts by CMS to obtain accurate cost data, one must acknowledge the weaknesses of using accounting data to reflect economic costs. For example, hospitals have discretion in the order in which they allocate the costs of non-revenue producing centers to service centers, which can change the relative costs of each service center. In addition, CMS gives specific instructions on the cost bases for allocating overhead costs (e.g. square footage for maintenance and repairs, or patient days for laundry services). These cost bases may not accurately reflect the actual balance of resource utilization across departments. Nevertheless, we have no reason to believe that accounting costs would be systematically higher (or lower) than economic costs for high volume versus low volume hospitals. Therefore, these errors are not likely to bias our regression estimates.

The ratio of cost to charges for each hospital as a whole may not provide an accurate correction for the cost of cardiac procedures at each hospital [30]. Measurement error in the dependent variable costs could lead to larger error variance when estimating the association between volume and costs, which would lower the chance of finding a significant association between these two variables. However, even with the potential for measurement error, we precisely estimate an association between higher procedure volume and lower unit costs. Moreover, past studies which have tried to adjust charges using department-level information have encountered wide variations in missing data and extreme outlier values across departments [31,32]. In addition, costs computed using an aggregate hospital cost-to-charge ratios were found generally to be within 10 percent of costs obtained from department-level cost data [33]. Another study found that profitability estimates (which require cost data) based on aggregate hospital cost-to-charge ratios were identical to estimates based on department-level data for Medicare and privately insured patients, and differed by only four percentage points for Medicaid patients [32]. Therefore, discrepancies between hospital-wide and department-specific cost-to-charge ratios are not likely to explain the differences in costs between low and high-volume hospitals identified in this study. Even if there were a large bias in our cost estimates–for example–if we underestimated the cost savings from regionalization by a factor of two, these cost savings would still be extremely small relative to the total costs of PCI and CABG in the US Further, several disease-specific cost analyses have relied on aggregate hospital cost-to-charge ratios rather than department-level data [34-36]. As one of these studies notes, analysis of a specific treatment with large fixed costs such as PCI and CABG yields a more homogeneous hospital sample, which mitigates any bias created by the use of hospital-level data [36]. In the absence of department-level financial data across multiple parts of the country and for all age groups, hospital-level cost-to-charge ratios are the best possible information source for making region-wide policy recommendations.

The hospital fixed effects control only for unobservable characteristics which are constant over time for a given hospital. It is possible that there are unobservable changes over time for a hospital that both increase its cardiac procedure volume and make the facility more efficient in the provision of care. Such unobservable changes would imply that we have underestimated the cost savings associated with regionalization. However, we are unaware of any literature identifying hospital ventures that have been associated with both lower costs and increased procedure volume for cardiac care. Unless such unobservable actions are widespread in our sample, they are unlikely to substantially bias our results.

Our dataset lacks information on physician costs. We are unaware of any studies which analyze the relation between hospital or physician procedure volume and physician costs. Even if higher hospital volume is associated with lower physician costs, the small fraction of patients now being treated in low-volume hospitals is still likely to lead to only limited cost savings under regionalization.

## Conclusion

As the costs of cardiac care continue to grow, clinicians and policy makers will be searching for solutions to control expenditures. With the presence of economies of scale in complex procedures, regionalization will be a likely candidate as a policy instrument. We find that regionalization based on recommendations that were made to reduce mortality will not be enough to significantly reduce health care costs. Also, due to limited availability of high-volume hospitals in certain geographic regions, regionalization policies may not always be feasible. Other studies have suggested that regionalization policies may produce underuse of clinically necessary cardiac procedures [37]. Therefore, regionalization policies require careful implementation to ensure that their potential benefits on patient outcome and costs are not outweighed by the hidden costs of barriers to access and underuse of needed services.

## Competing interests

The author(s) declare that they have no competing interests.

## Authors' contributions

VH carried out the acquisition of data, performed the statistical analysis, and drafted the manuscript. LAP critically reviewed the manuscript for important intellectual data. Both authors participated in the conception and design of the study, analysis and interpretation of data, and have read and approved the final manuscript.
